# CDX2 hox gene product in a rat model of esophageal cancer

**DOI:** 10.1186/1756-9966-28-108

**Published:** 2009-08-07

**Authors:** Giuseppe Ingravallo, Luigi Dall'Olmo, Daniela Segat, Matteo Fassan, Claudia Mescoli, Emanuela Dazzo, Carlo Castoro, Lorenzo Polimeno, Christian Rizzetto, Maurizio David Baroni, Giovanni Zaninotto, Ermanno Ancona, Massimo Rugge

**Affiliations:** 1 Department of Medical Diagnostic Sciences & Special Therapies, Pathology Unit, University of Padova, Padova, Italy; 2 Department of Pathological Anatomy, University of Bari, Bari, Italy; 3 Istituto Oncologico Veneto (IOV-IRCCS), Padova, Italy; 4 Department of Mathematics, Physics, and Natural Sciences, University of Padova, Padova, Italy; 5 Department of Emergency & Organ Transplantation, University of Bari, Bari, Italy; 6 Department of Gastrointestinal & Surgical Sciences, Clinica Chirurgica III, University of Padova, Padova, Italy; 7 Department of General Surgery, Sts Giovanni & Paolo Hospital, Venezia, Italy

## Abstract

**Background:**

Barrett's mucosa is the precursor of esophageal adenocarcinoma. The molecular mechanisms behind Barrett's carcinogenesis are largely unknown. Experimental models of longstanding esophageal reflux of duodenal-gastric contents may provide important information on the biological sequence of the Barrett's oncogenesis.

**Methods:**

The expression of *CDX2 *hox-gene product was assessed in a rat model of Barrett's carcinogenesis. Seventy-four rats underwent esophago-jejunostomy with gastric preservation. Excluding perisurgical deaths, the animals were sacrificed at various times after the surgical treatment (Group A: <10 weeks; Group B: 10–30 weeks; Group C: >30 weeks).

**Results:**

No Cdx2 expression was detected in either squamous epithelia of the proximal esophagus or squamous cell carcinomas. *De novo *Cdx2 expression was consistently documented in the proliferative zone of the squamous epithelium close to reflux ulcers (Group A: 68%; Group B: 64%; Group C: 80%), multilayered epithelium and intestinal metaplasia (Group A: 9%; Group B: 41%; Group C: 60%), and esophageal adenocarcinomas (Group B: 36%; Group C: 35%). A trend for increasing overall Cdx2 expression was documented during the course of the experiment (*p *= 0.001).

**Conclusion:**

*De novo *expression of Cdx2 is an early event in the spectrum of the lesions induced by experimental gastro-esophageal reflux and should be considered as a key step in the morphogenesis of esophageal adenocarcinoma.

## Background

In the homeobox gene family, the caudal-related *CDX2 *gene encodes for an intestine-specific transcription factor involved in both cell turnover and intestinal differentiation [1]. Nuclear immunostain for Cdx2 is restricted to the native intestinal epithelia and its *de novo *expression is considered as suitable marker of a newly achieved intestinal commitment [2,3].

Barrett's esophagus (BE) is defined as replacement of the native esophageal squamous epithelium by columnar (intestinalized) mucosa [4-6]. Longstanding exposure of the squamous esophageal epithelium to gastric reflux is a primary risk factor for columnar metaplasia, which is consistently considered as precursor of esophageal adenocarcinoma (Ac) [7,8].

Esophageal Ac is the final step in a sequence of phenotypic changes that include long-standing esophagitis, columnar cell metaplasia, and non-invasive neoplasia (NiN). The molecular derangements occurring in each of these phenotypic changes are largely unknown and they involve both genetic and chromosomal instability [9,10]. More information on such molecular changes is crucial in any strategy of primary prevention of Barrett's Ac [11-14].

In humans, both practical and ethical limitations prevent any sequential exploration of the cascade of Barrett's Ac, so experimental models are used to characterize the biological alterations leading to neoplastic transformation [15-31].

In this experimental study, the expression of Cdx2 protein was tested over the whole spectrum of phenotypic lesions detected in a surgical murine model of esophago-gastroduodenal anastomosis (EGDA) resulting in longstanding esophageal reflux of gastro-duodenal contents [19,21-24,29].

## Methods

### Experimental design

**Figure 1:**
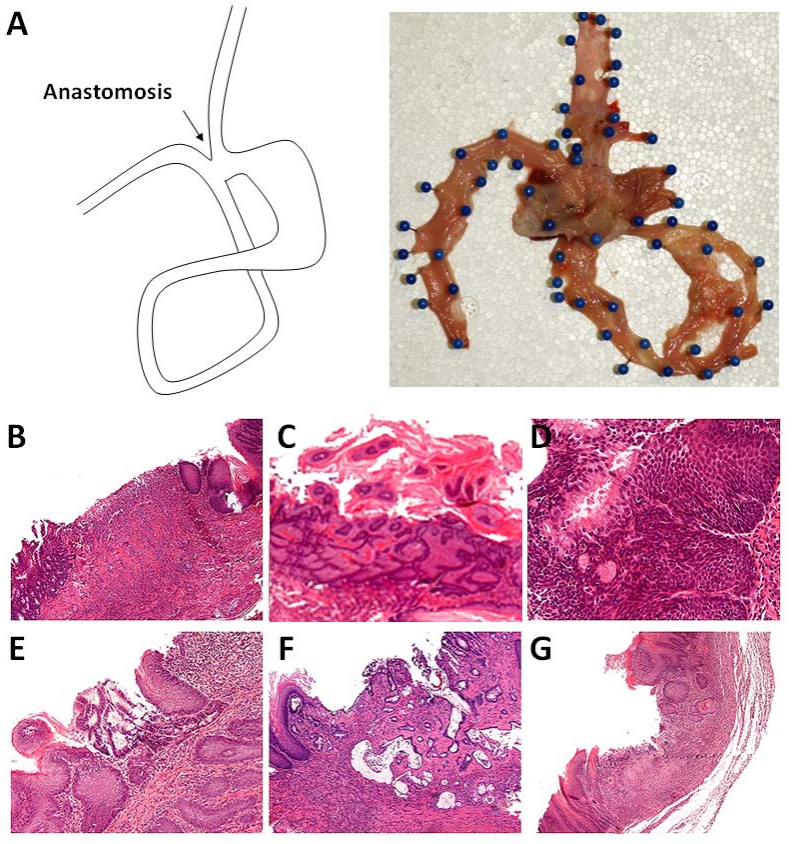
**Pathology findings of the esophageal cancer model**. (A) Schematic illustration of the surgical intervention of the Kumagai-Hattori model (*left*) and representative macroscopic picture (right): unfixed esophagus, stomach and jejunum (excised en bloc) are opened through the dorsal wall (mucosal surface upward). (B-G) Histological findings observed (H&E staining): (B) anastomosis ulcer; (C) squamous cell polypoid hyperplasia; (D) multilayered epithelium; (E) specialized columnar epithelium (intestinal metaplasia); (F) adenocarcinoma; (G) squamous cell cancer. (Original magnifications, 40×, 20× and 10×)

An esophago-gastroduodenal anastomosis was performed on 74 eight-week-old male *Wistar Han *rats (Charles River, Lecco, Italy), as described elsewhere [19,21-24,29]. Before surgery, the animals were kept under standard laboratory conditions. In brief, a 1.5 cm side-to-side surgical EGDA was created between the first duodenal loop and the gastro-esophageal junction, about 3 cm distal to Treitz's ligament, with accurate mucosa-to-mucosa opposition (Figure 1), so that duodenal and gastric contents flowed back into the esophagus. Unlike other models, this "Kumagai-Hattori" model preserves the animal's normal stomach function and nutritional status [19,21,22].

Postoperatively, the animals had free access to water and food. No treatments with any known carcinogen were applied.

Ten of the 74 rats died (mainly of respiratory complications) within 7 days after surgery and were not considered. As in already published experimental models, the animals were sacrificed at different times after surgery (i.e. Group A [22 rats] after <10 weeks [range = 3–9.9], Group B [22 rats] after 10–30 weeks [range = 10–29.7], and Group C [20 rats] after >30 weeks [range = 31–54]) [19,21,22,27,28].

This study was approved by the Institutional Animal Care Committee of the University of Padova. All procedures were performed in accordance to the Italian law on the use of experimental animals (DL n. 116/92 art. 5) and according to the "Guidelines on the Care and Use of Laboratory Animals" (NIH publication 85–93, revised in 1985).

### Pathology

Immediately after death, the thoracic and abdominal cavities were examined and the esophagus, stomach, and jejunum were excised *en bloc*. The esophagus was opened longitudinally through the dorsal wall. With the mucosal surface uppermost, the margins of the specimen were fixed to a cork plate with pins. Gross specimens were fixed in 10% neutral-buffered formalin for 24 hours. All specimens were examined grossly (see gross pathology) and cut serially (2–3 mm thick coronal sections). The tissue samples were routinely processed. Tissue sections 4 μm thick were obtained from paraffin blocks and stained with Haematoxylin & eosin. Lung, liver, kidney and spleen tissues were also collected for histological assessment. Two experienced gastrointestinal pathologists (GI & MF) reviewed all the slides.

**Figure 2:**
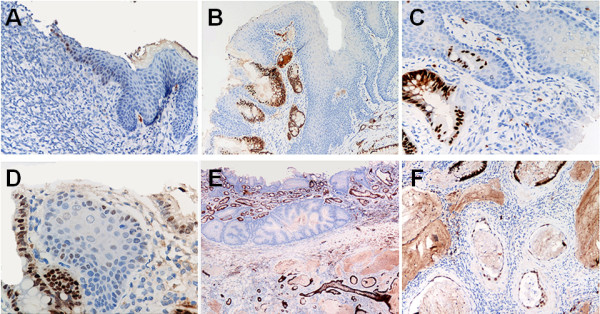
**CDX2 immunohistochemical expression**. (A) Cdx2 aberrant nuclear expression in the basal layer of the squamous native esophageal epithelium close to mucosal erosion. (B-C) Strong Cdx2 nuclear immunostain in multilayered epithelium and intestinalized columnar epithelium. (D) Strong Cdx2 expression in intestinal metaplasia and aberrant Cdx2 expression in basal squamous cells of native esophageal epithelium. (E-F) Strong Cdx2 positivity in two cases of esophageal adenocarcinoma. Note in E, the contrast with the Cdx2 negative native esophageal epithelium. (Original magnifications, 40×, 20× and 10×)

Histological findings in the squamous epithelium lesions were grouped into 5 main categories (Table 1, Figure 2) [16,18,25]: (1) non-ulcerative esophagitis; (2) ulcers (always associated with inflammation and granulation tissue); (3) regenerative-hyperplastic (also polypoid) lesions; (4) multilayered epithelium (MLE) and/or intestinal metaplasia within squamous epithelium; and (5) carcinomas (distinguishing esophageal adenocarcinoma [Ac] from squamous cell esophageal cancer [SCC]).

**Table 1:** Histological findings and Cdx2 expression in the rat model of esophageal carcinogenesis.

Histology		Cdx2 expression	Group A (<10 weeks, n = 22)	Group B (10–30 weeks, n = 22)	Group C (>30 weeks, n = 20)
			
			cases (%)	cases (%)	cases (%)
Non-ulcerative esophagitis		-	22/22 (100.0%)	22/22 (100.0%)	20/20 (100.0%)
Inflammatory-ulcerative lesions		+	15/22 (68.2%)	14/22 (63.6%)	16/20 (80.0%)
Regenerative-hyperplastic lesions		+	10/22 (45.5%)	8/22 (36.4%)	10/20 (50.0%)
Metaplastic lesions	IM	+	2/22 (9.1%)	9/22 (40.9%)	12/20 (60.0%)
	MLE				
Carcinomas	Ac	+	0/22 (0.0%)	8/22 (36.4%)	7/20 (35.0%)
	SCC	-	0/22 (0.0%)	2/22 (9.1%)	2/20 (10.0%)

Note: n = number of cases; wks = weeks; IM = intestinal metaplasia; MLE = multilayered epithelium; Ac = adenocarcinomas; SCC = squamous cell carcinomas.

Non-ulcerative esophagitis was defined as sub-epithelial inflammatory infiltrate, generally coexisting with intraepithelial leukocytes; epithelial micro-erosions were arbitrarily included in this category.

Ulcers (defined as the complete loss of the mucosal layer with muscle exposure) always coexisted with granulation tissue and hyperplastic-regenerative changes of the surrounding epithelium.

Hyperplastic lesions were defined as thickening of the squamous epithelium (sometimes hyperkeratotic) with no cellular atypia. Regenerative lesions were assessed in terms of the increased length of the papillae in the lamina propria (>70% of mucosal thickness), also coexisting with hyperplasia of the proliferative compartment (>20% of the mucosal thickness) [16,18,25].

Metaplastic intestinalization was defined as the presence of both columnar epithelia and goblet cells [16,18,25]. Multilayered epithelium (MLE) is a hybrid epithelium in which both squamous and columnar epithelia coexist ("protometaplasia"); consistently with its phenotype, MLE expresses cytokeratins of both squamous and columnar differentiation [32].

Cancers were distinguished according to their histotype. Squamous cell carcinoma consisted in a neoplastic growth of squamous epithelia with different grades of differentiation. Adenocarcinoma consisted of atypical tubular/cystic glands with abundant extra-cellular mucins (Figure 1). Consistently with previous studies [18,27,29], we did not consider an autonomous group of "atypical" epithelial lesions. In fact, such phenotypical alterations are inconsistently described by the current international literature and their negligible prevalence in our study represents the rationale of including them among non-cancer lesions.

### Immunohistochemistry (IHC)

Cdx2 immunostain (anti-mouse-Cdx2 antibody, dilution 1:10; BioGenex Laboratories Inc., San Ramon, CA) was applied on 4-μm tissue sections. In all cases, a standardized ABC method was used, implemented on the Ventana Benchmark XT system (Touchstone, AZ). Appropriate positive (mouse colon) and negative (mouse spleen) controls were always run concurrently.

Cdx2 IHC expression was assessed negative (no immunostaining or sparse Cdx2-stained nuclei in less than 5% of the cells) or positive (nuclear immunoreaction in 5% or more of the cells).

### Statistical analysis

Differences seen during the course of the experiment in terms of the incidence of pre-neoplastic/neoplastic lesions and/or overall Cdx2 staining (defined as the percentage of Cdx2-positive cases amongst the different histological categories) were evaluated using the modified Kruskal-Wallis non-parametric test for trend.

Differences were considered statistically significant when *p *< 0.05. All statistical analyses were performed with STATA software (Stata Corporation, College Station, Texas).

## Results

### Pathology (gross and histology)

Three main types of gross lesion were encountered, *i.e*. reddened flat mucosa (at both gastric and esophageal sites), ulcers, and protruding and/or nodular lesions. The red mucosa was seen in the esophagus proximal to the EGDA (proximal stomach and distal esophagus), whereas both ulcers and protruding and/or nodular lesions were always located close to the anastomosis. All gross abnormalities were sampled for histological assessment.

The histological lesions detected in the 3 groups of animals are summarized in Table 1 and Figure 1. All rats had reflux (erosive or non-erosive) esophagitis proximal to the anastomosis. Mucosal ulcers were located in the middle/lower thirds of the esophagus in 15/22 (68.2%) animals in Group A; 14/22 (63.6%) in Group B and 6/20 (30%) in Group C. Regenerative/hyperplastic changes were also identified (Group A = 10/22 [45.5%]; Group B = 8/22 [36.4%], Group C = 10/20 [50.0%]).

None of the animals in Group A revealed any intestinal metaplasia (IM) and only 2 cases of MLE were seen (9.1%; both located close to the EGDA). In Groups B and C, MLE and IM were consistently identified and their prevalence increased significantly with the time elapsing after the operation (and with a similar prevalence of IM and MLE): Group B = 9/22 (40.9%); Group C = 12/20 (60.0%) (test for trend, *p *= 0.001).

Esophageal cancers were only documented histologically more than 10 weeks after the operation (no cancers came to light in Group A). In Group B, there were 10 esophageal malignancies (45.5%; 8 esophageal Ac and 2 SSC); in Group C, 9 cases of cancer were detected (45.0%; 7 esophageal Ac and 2 SSC). Eight cases of esophageal Ac were located proximally to the cardia; both cases of SSC developed in the middle-cervical esophagus. No neoplastic vascular invasion or metastatic lesions (nodal or extranodal) coexisted with the invasive cancers.

### Cdx2 expression

The prevalence of Cdx2 nuclear expression in each of the histological categories considered is shown in Table 1 and Figure 2. Cdx2 was never expressed in native squamous epithelia (including any non-ulcerative esophagitis) in the upper third of the esophagus. Aberrant and inconsistent Cdx2 nuclear expression was seen in the proliferative compartment of the squamous mucosa, close to esophageal ulcers and/or hyperplastic lesions (Group A = 4/22 [18.2%]; Group B = 6/22 [27.3%]; Group C = 8/20 [40.0%]).

In Groups B and C, intestinal metaplasia, multilayered epithelium, and esophageal Ac all consistently showed Cdx2 expression (Cdx2+ve cases: IM = 21/21; MLE = 21/21; Esophageal Ac = 15/15). A trend towards higher levels of overall Cdx2 expression was documented during the course of the experiment (test for trend; *p *= 0.001). None of the 4 cases of SCC showed Cdx2 staining.

## Discussion

Gastro-esophageal reflux is generally considered the main promoter of esophageal columnar metaplasia and adenocarcinoma.

Cdx2 is a transcription factor that regulates the expression of differentiation-related molecules and it is specifically involved in intestinal cells commitment. Based on this rationale, Cdx2 immunohistochemical expression was explored in a rat model of EGDA.

As in previous studies, *de novo *Cdx2 expression was documented in the whole spectrum of phenotypic changes induced by experimental EGDA. The prevalence of Cdx2 expression increased significantly with time (*i.e*. the prevalence of IM and MLE was higher in Groups B and C than in Group A), suggesting a time-dependent relationship between the "chemical" injury and the severity of the lesions.

Cdx2 expression in full-blown metaplastic transformation was expected. This study, however, also showed that *de novo *Cdx2 expression is an early event among the morphological changes caused by the refluxate. The early deregulation of Cdx2 expression has already been demonstrated by Pera *et al*. [28], who described Cdx2 immunostaining in the basal cell layer close to esophageal ulcers 16 weeks after surgery. More recently, however, in a study using a similar EGDA model, Xiaoxin Chen *et al*. [17] considered Cdx2 over-expression as a late marker of the metaplastic cascade.

Our study provides evidence that "protometaplastic" changes (in both the squamous stem cell and MLE) could be revealed by Cdx2 immunostaining even before the IM becomes histologically assessable. It is worth noting that MLE (which can also be a feature of normal rat mucosa) might be considered as a "partially-committed" cell population, prone to a chimeric intestinal differentiation under critical conditions (such as those produced by EGDA). Such speculations might also apply to the staminal cells compartment of the native esophageal mucosa: in cultured esophageal epithelia, in fact, chemical injuries (acid and/or bile components) may result in *Cdx2 *promoter demethylation/activation [33]. These hypotheses are further supported by the finding that no Cdx2 expression was detected in squamous epithelia (far from esophageal ulcers/metaplastic changes), nor in any of the 4 cases of SCC.

Together with Cdx2, also other intestine-specific transcription factors have been described as involved in Barrett's epithelium development [34-36]. In a similar rat model, Kazumori *et al*. [36] showed, that a de novo expression of Cdx1 (another member of the caudal-related homeobox gene family) significantly antecedes Cdx2 expression [35,36]. Further studies are needed to investigate on the interplay of these two genes in the morphogenesis of Barrett's mucosa.

The SCC cases detected in this study prompts us to hypothesize that the environmental conditions resulting from EGDA may also result into the derangement of cell regulatory mechanisms involving both multilayered and squamous epithelia. Previous studies documented that several transcription factors (p63, among others) are over-expressed in squamous esophageal epithelia after EGDA. Such an observation could explain, at least in part, the high prevalence of SCC documented in this and other studies.

## Conclusion

In conclusion, the Kumagai-Hattori model of esophago-gastroduodenal anastomosis (with gastric preservation) is an useful *in vivo *model of esophageal carcinogenesis. Both the stem cell compartment and the multilayered epithelium are early involved in the metaplastic intestinalization of the native esophageal mucosa.

## Competing interests

The authors declare that they have no competing interests.

## Authors' contributions

All authors of this research paper have directly participated in the planning, execution, or analysis of the study. All authors read and approved the final manuscript.
